# Impact of Pulsed Electric Fields and pH on Enzyme Inactivation and Bioactivities of Peptic Hydrolysates Produced from Bovine and Porcine Hemoglobin

**DOI:** 10.3390/foods11213313

**Published:** 2022-10-22

**Authors:** Zain Sanchez-Reinoso, Sarah Todeschini, Jacinthe Thibodeau, Laila Ben Said, Ismail Fliss, Laurent Bazinet, Sergey Mikhaylin

**Affiliations:** 1 Institute of Nutrition and Functional Foods (INAF), Université Laval, Quebec City, QC G1V 0A6, Canada; 2 Laboratory of Food Sustainability (EcoFoodLab), Food Science Department, Université Laval, Quebec City, QC G1V 0A6, Canada; 3 Laboratoire de Transformation Alimentaire et Procédés Électromembranaires (LTAPEM, Laboratory of Food Processing and Electro-Membrane Processes), Food Science Department, Université Laval, Quebec City, QC G1V 0A6, Canada; 4 International Associated Laboratory in Bioproduction of Natural Antimicrobials (LIAAN), Université Laval (Canada) and Lille University (France), Quebec City, QC G1V 0A6, Canada

**Keywords:** pulsed electric fields, blood valorization, hemoglobin, bioactive peptides, pepsin

## Abstract

The production of bioactive peptides from hemoglobin via peptic hydrolysis is a promising alternative to valorizing slaughterhouse blood proteins. Nevertheless, it has some limitations such as low yield, high cost of enzymes, and the use of chemical reagents. The latter is aggravated by the pH increase to inactivate the enzyme, which can affect the bioactivity of the peptides. Thus, this study aimed to evaluate the effect of pulsed electric fields (PEF) on the pepsin inactivation and biological activities (antimicrobial and antioxidant) of hemoglobin hydrolysates. Bovine (Hb-B) and porcine (Hb-P) hemoglobin were hydrolyzed with pepsin for 3 h and treated with PEFs to inactivate the enzyme. The degree of hydrolysis (DH) did not show significant changes after PEF inactivation, whereas peptide population analysis showed some changes in PEF-treated hydrolysates over time, suggesting residual pepsin activity. PEF treatments showed no significant positive or negative impact on antimicrobial and antioxidant activities. Additionally, the impact of pH (3, 7, and 10) on bioactivity was studied. Higher pH fostered stronger anti-yeast activity and DPPH-scavenging capacity, whereas pH 7 fostered antifungal activity. Thus, the use of hemoglobin from the meat industry combined with PEF treatments could fit the circular economy concept since bioactive peptides can be produced more eco-efficiently and recycled to reduce the spoilage of meat products. Nevertheless, further studies on PEF conditions must be carried out to achieve complete inactivation of pepsin and the potential enhancement of peptides’ bioactivity.

## 1. Introduction

Slaughterhouse blood remains a serious management problem for the meat industry due to the large volume produced, its low added value, as well as its high pollutant load and disposal costs [1]. Its solid fraction (red blood cells) contains about 90% hemoglobin [2], a tetrameric protein comprising four globin subunits bound by four heme groups (two α-globins + two β-globins + four hemes), which has been widely described as a proper source of bioactive peptides with antihypertensive [3,4], opioid [5], antihyperglycemic [6], antimicrobial [4,7,8,9,10,11,12], and antioxidant [13,14,15] activities. Particularly, hemoglobin-derived antimicrobial and antioxidant peptides have gained great interest in the meat industry since they could be reused for the protection of meat and meat products (e.g., ham and sausages) against microbial spoilage and lipid oxidation under a circular economy approach, as was demonstrated for bovine hemoglobin [7]. Those bioactive peptides are commonly produced via enzymatic hydrolysis. In this context, pepsin has shown better performance for hemoglobin hydrolysis in comparison to other enzymes such as trypsin, papain, and alcalase, leading to a higher production of bioactive peptides [3,13,14,16]. More than 30 antimicrobial peptides have been identified in peptic hydrolysates of bovine hemoglobin (Hb-B); among them, antibacterial activity against both Gram^+^ and Gram^−^ strains, as well as antifungal (*Candida albicans*, *Aspergillus niger*, *Mucor racemosus*, *Penicillium crustosum*, and *Paecilomyces* spp) and anti-yeast (*Rhodotorula mucilaginosa*) activities have been reported [17]. In contrast, antimicrobial peptides derived from porcine hemoglobin (Hb-P) have been studied to a much lesser extent, but their antibacterial (*Micrococcus luteus*, *Escherichia coli*, *Salmonella Newport*, and *Listeria ivanovii*) antifungal (*Mucor racemosus*), and anti-yeast (*Rhodotorula mucilaginosa*) activities have also been demonstrated in crude peptic hydrolysates [17,18]. Regarding hemoglobin-derived antioxidant peptides, their specific sequences have not been extensively characterized. Only the antioxidant activity of the peptide α(137–141), which presents the same sequence for Hb-B and Hb-P, as well as β(115–129) [19] derived from Hb-P, has been verified. However, the antioxidant activity of crude hydrolysates [13,18,20,21] and multiple peptide fractions recovered via ultrafiltration [13,19], gel filtration chromatography [22], and electrodialysis with bipolar membrane [23] support the presence of antioxidant peptides in hemoglobin hydrolysates.

Nevertheless, the approach of bioactive peptide production via enzymatic hydrolysis has some limitations to be solved. The conventional production of bioactive peptides from hemoglobin includes native hemoglobin denaturation, pH adjustment during pepsin hydrolysis, the cessation of the enzymatic reaction, and sometimes, an additional step of the discoloration of hydrolysates to avoid organoleptic changes in food formulations. Hence, a big number of chemical reagents for acidification and basification is involved in those stages [23,24]. Furthermore, pepsin inactivation is usually performed by increasing the pH (pH > 8.0), which can affect the net charge of peptides and, consequently, their bioactivity [25]. These aspects make it difficult to achieve eco-friendly development of this process. Therefore, improving the performance of this process to achieve a sustainable application continues to be a major research challenge.

Among the emergent green technologies for protein valorization, pulsed electric fields (PEFs) have become a promising alternative. They consist of applying intermittent electrical fields (<3000 Hz) at moderate–high field strength (0.1–50 kV/cm) for a short time (µs-ms) [26,27]. This technology allows modification of the physicochemical properties of proteins by affecting the protein tridimensional structural elements due to the interaction between the electrical field and their dipole moments, as well as the ionization of the carboxylic groups (-COO^−^), amino groups (-NH_3_^+^), and radical groups of electrically charged residues (e.g., lysine, arginine, histidine, aspartic acid, and glutamic acid) [28]. Hence, PEFs offer a broad range of applications such as promoting the enzymatic hydrolysis of proteins, enzyme activation or inactivation, the improvement of protein techno-functional properties, and the biological activity enhancement of peptides [26,28,29,30,31]. Regarding enzyme inactivation, PEFs can alter the tertiary and secondary structure of enzymes, and plays an important role in enzyme stability involving weak non-covalent interactions such as hydrogen bonds and hydrophobic interactions [31]. Several studies have supported the inactivation of enzymes such as peroxidases [32,33,34], lipoxygenases [35,36,37], alkaline phosphatases [38,39], and papain [40,41], among others, via PEF treatment. In the case of pepsin, Yang et al. [42] and Zhao and Yang [43] reported that pepsin in aqueous solutions can be inactivated by PEFs with electric-field strengths between 20–40 kV/cm, which was associated with the loss of β-sheet structure in the pepsin molecule. However, to the best of our knowledge, no studies have been reported on the use of PEF treatments as an alternative to inactivate pepsin or other proteases during the production of bioactive peptides via the enzymatic hydrolysis of hemoglobin.

In addition, the use of PEFs in antioxidant activity enhancement has been reported in peptides derived from several sources, including egg-white protein [44,45], soybean protein [46], corn gluten [47], pine nut [46,48], shrimp protein [49], etc. An increase from 48.2% to 62.6% in the DPPH-scavenging capacity of egg-white peptides (10–30 kDa) treated with PEFs (10 kV/cm and 2400 Hz) was reported by Wang et al. [44], whereas the same peptide fraction treated with 10 kV/cm and 2000 Hz led to a FRAP value increase of 44.2% compared to the untreated peptides [45]. Furthermore, Wang et al. [47] increased the DPPH-scavenging capacity by 32.1% in a solution of 10 mg/mL corn peptides (10 to 30 kDa) by applying an electric-field strength of 15 kV/cm and pulse frequency of 2000 Hz. The enhancement of antioxidant activity on specific peptides has also been studied. For instance, Zhang et al. [49] evaluated the antioxidant activity PEF improvement of the peptide QMDDQ obtained from shrimp protein. They observed that PEF treatment with 40 kV/cm and 4000 Hz showed the best antioxidant activity, which may be due to the fact that PEFs transform the β-sheet into a random coil which can inhibit the steric hindrance of free radicals. However, the use of PEFs in improving the antioxidant activity of hemoglobin hydrolysates is an unexplored domain to date. Additionally, it is important to note that no studies have been performed on the impact of PEF treatments on the antimicrobial activity of peptides. Similarly, there is a lack of studies related to the impact of pH on peptide bioactivity. Most studies related to the bioactivity of hemoglobin-derived peptides via peptic hydrolysis have reported a basic pH (>8.0 for pepsin inactivation) or have not specified the hydrolysate pH at which the bioactivity tests were performed [7,8,11,14]. Nevertheless, pH affects the charge state of amino acids and functional groups of peptides, and consequently, their physicochemical properties and bioactivities. Hence, it plays an important role in their biological activities such as electrostatic interactions with anionic surfaces during microbial inactivation (antimicrobial peptides) and free-radical scavenging (antioxidant peptides) [25,50]. For this reason, the impact of pH on peptides is a major point to study since this parameter can be used to achieve a certain targeting effect in practical applications.

Thus, the main objectives of this study were (1) to evaluate the impact of PEFs on pepsin inactivation in hydrolysates of bovine and porcine hemoglobin, (2) to characterize the peptide populations after pepsin inactivation via PEF treatment, and (3) to evaluate the effects of PEF treatment and pH value on the antimicrobial and antioxidant activities of the final hydrolysates.

## 2. Materials and Methods

### 2.1. Materials

Bovine and porcine hemoglobin (purity > 98%) were purchased from Sigma-Aldrich Chemical Corporation (St. Louis, MO, USA), as was pepsin from porcine gastric mucosa (EC 3.4.23.1, 3200–4500 units/mg protein). Pepsin activity was confirmed using the enzymatic assay suggested by the same supplier [51]. HCl and NaCl used in hydrolysis were purchased from Fisher Scientific (Nepean, ON, Canada). Sodium dodecyl sulfate (SDS, Bio-Rad Laboratories Inc, Japan), sodium tetraborate (Fisher Scientific, Ottawa, ON, Canada), *o*-phthalaldehyde (OPA, Sigma-Aldrich, St. Louis, MO, USA), DL-leucine (Alfa Aesar, Shanghai, China), and β-mercaptoethanol (Sigma-Aldrich, St. Louis, MO, USA) were used for determination of the degree of hydrolysis. All reagents used in the RP-UPLC-MS/MS analyses were of analytical grade and purchased from Sigma-Aldrich. Fluorescein, 1,1-diphenyl-2-picrylhydrazyl (DPPH), 2,2′-azobis(2-methylpropionamidine) dihydrochloride (AAPH), and (±)-6-Hydroxy-2,5,7,8-tetramethylchromane-2-carboxylic acid (Trolox) used in antioxidant tests were also purchased from Sigma-Aldrich Chemical Corporation (St. Louis, MO, USA), whereas ethanol was purchased from Commercial Alcohols Inc. (Brampton, ON, Canada) and sodium phosphate from Fisher Scientific (Ottawa, ON, Canada). Potato dextrose broth (PDB, DIFCO™, Sparks, MD, USA), tryptic soy broth (TSB, BD Bacto™, Franklin Lakes, NJ, USA), agar (Criterion, Hardy Diagnostics, Santa Maria, CA), dichloran–rose bengal–chloramphenicol agar (DRBC agar, DIFCO™, Sparks, MD, USA), peptone (BD Bacto™, Sparks, MD, USA), ampicillin (Sigma-Aldrich, St. Louis, MO, USA), and natamycin (Sigma-Aldrich, St. Louis, MO, USA) were employed for the antimicrobial tests.

### 2.2. Configuration of PEF System

**Figure 1:**
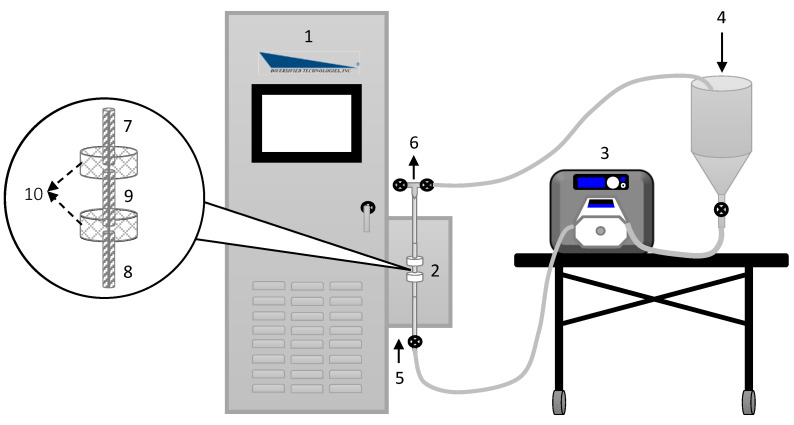
Schematic diagram of the experimental setup for pepsin inactivation by PEFs in hemoglobin hydrolysates: (1) modulator cabinet with display control, (2) treatment chamber, (3) peristaltic pump, (4) feed container, (5) solution inlet, (6) solution outlet, (7 and 8) low-voltage electrodes, (9) high-voltage electrode, and (10) ceramic isolators.

A schematic diagram of the experimental assembly is displayed in Figure 1. A Diversified Technologies Power Mod™ 25 kW Pulsed Electric Field System (Diversified Technologies Inc., Bedford, MA, USA) was used, which consisted of the PEF treatment enclosure and a modulator cabinet. The PEF treatment enclosure comprised a single co-linear treatment chamber with two treatment zones, consisting of three stainless steel electrodes (a high-voltage electrode and two low-voltage electrodes connected to the ground) and two ceramic isolators. The electrode diameter was 6 mm, and the gap distance was 8 mm. The PEF system supplied monopolar pulses with a square shape and a pulse width between 1 and 10 μs. The PEF system was fed with a Masterflex^®^ I/P Digital Drive pump with an Open-Head sensor (HV-77420-18, Cole Parmer, Vernon Hills, IL, USA) equipped with a Masterflex^®^ I/P Easy-Load pump head (HV-77602-30, Cole Parmer, USA) with Masterflex^®^ I/P 73 laboratory tubing (ZN-96419-73, Cole Parmer, USA). All the experiments were performed via recirculation of the solution to achieve a desired number of applied pulses.

### 2.3. Experimental Protocol

**Figure 2:**
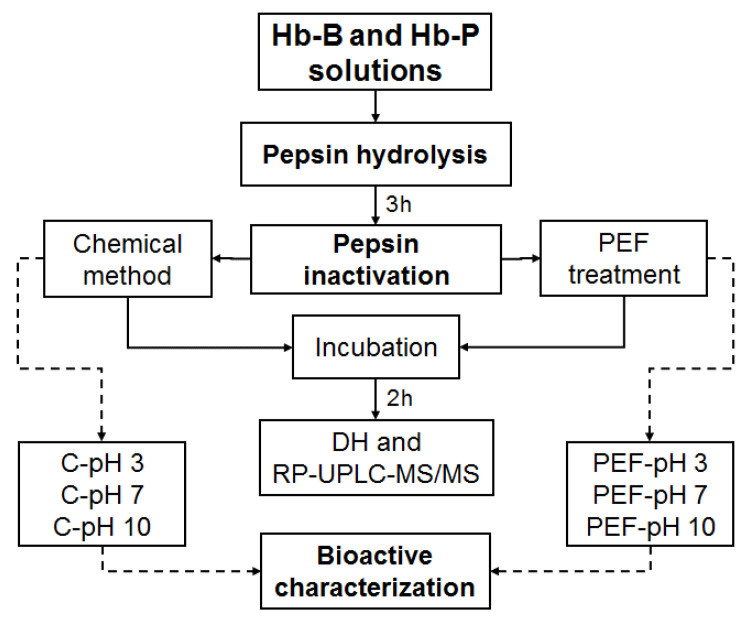
Experimental workflow and analyses (DH: degree of hydrolysis, PEF: pulsed electric fields, Hb-B and Hb-P: bovine and porcine hemoglobin, respectively).

The impact of PEF treatment on the inactivation of pepsin and the biological activity of Hb-B and Hb-P hydrolysates was evaluated. Figure 2 shows the workflow of the present study. Hb-B and Hb-P hydrolysates were produced according to Sanchez-Reinoso et al. [17] with slight modifications. Briefly, 0.5% (*w*/*w*) solutions (1 L) of Hb-B and Hb-P were prepared with Milli-Q water via overnight stirring (150 rpm) at 4 °C. The pH of the solutions was adjusted at 3 with 2 M HCl and incubated in a shaking water bath (VWR International, LLC, Radnor, PA, USA) at 37 °C for 15 min with agitation (80 rpm). Then, pepsin was added at an enzyme/substrate ratio of 1:11 mol/mol. Peptic hydrolysis was carried out for 3 h with constant temperature, pH, and agitation (37 °C, pH 3, and 80 rpm).

After 3 h of hydrolysis, the Hb-B and Hb-P hydrolysates were immediately treated with PEFs to inactivate pepsin. A total of 73 pulses were applied with an electric field of 23.8 kV/cm, which were achieved using a pulse width of 10 μs, a frequency of 90 Hz, and a constant flow rate of 100 mL/min with recirculation for 30 min. Those parameters were determined via preliminary assays. The electrical conductivity of hemoglobin hydrolysates was measured using a benchtop conductivity meter (Orion star A212, Thermo Scientific, Chelmsford, MA, USA), which was 1064.5 ± 8.5 µS/cm for Hb-B hydrolysates and 1052.8 ± 13.6 µS/cm for Hb-P hydrolysates. After PEF treatment, the solutions were collected, and 100 mL of samples were put back in the water bath at 37 °C for 2 h to evaluate enzyme inactivation performance. No significant change in the pH of the hydrolysates was observed after PEF treatment, which remained at 3.07 ± 0.7 in both the Hb-B and Hb-P hydrolysates. Samples (5 mL) were collected at 3, 3.5, 4, 4.5, and 5 h for further characterization of the degree of hydrolysis (DH) and peptide population via RP-UPLC-MS/MS. In this case, the hydrolysis of all collected samples was stopped by raising the pH to 10 by using 2 M NaOH. The remaining hydrolysate solution (~900 mL) was divided into three equal parts and their pH values were adjusted to 3, 7, and 10 to evaluate the impact of pH on the antimicrobial and antioxidant activities of the final hydrolysate (3 h). Control samples untreated with PEFs (chemical method in Figure 2) at different pH values (3, 7, and 10) were also prepared for both Hb-B and Hb-P. In this case, the pH adjustment was carried out by first increasing the pH value to 10 to inactivate pepsin, and then, reducing it to 7 and 3. The samples were prepared in triplicate.

### 2.4. Determination of the DH

The DH of the hydrolysates was determined via *o*-phthalaldehyde (OPA) assay according to Church et al. [52] with slight modifications. The OPA reagent was prepared by mixing 100 mL of 100 mM sodium tetraborate, 10 mL of 20% *w*/*w* sodium dodecyl sulfate (SDS), 4 mL of OPA/methanol solution (40 mg/mL), 400 μL of β-mercaptoethanol, and distilled water to a total volume of 200 mL. An aliquot of 150 µL of each hydrolysate and 3 mL of OPA reagent were transferred into a 4.5 mL acrylic cuvette (Fisher Scientific, Pittsburgh, PA, USA). The samples were mixed via inversion and incubated at room temperature for 2 min. Then, the absorption at 340 nm was measured in an Agilent 8453 UV–visible spectroscopy system (Hewlett-Packard Company, Waldbronn, Germany) and the number of free amino groups was determined with reference to a DL-leucine standard curve (0.75–3 mM DL-leucine, R^2^ ~0.999). The DH was calculated using Equation (1) and reported as an average value from three repetitions.

(1) $$\mathrm{DH}\;=\frac{\left(h-h_{0}\right)}{h_{\mathrm{tot}}}\times 100$$

where DH (%) is the degree of hydrolysis, h (mEq/g protein) is the number of free amino groups released via the cleavage of peptide bonds during the peptic hydrolysis of hemoglobin, h_0_ (mEq/g protein) is the number of free amino groups of the substrate (hemoglobin) before the addition of pepsin, and h_tot_ (mEq/g protein) is the total number of peptide bonds for hemoglobin, which was 8.3 mEq/g protein [53].

### 2.5. Peptide Identification and Quantification by RP-UPLC-MS/MS

The peptide population of the hydrolysates was studied to confirm pepsin inactivation since a change in peptides after PEF treatment could indicate that the enzyme reaction continued. For this purpose, both treated and untreated hydrolysates of Hb-B and Hb-P were analyzed via RP-UPLC as described by Abou-Diab et al. [23], with slight modifications. A 1290 Infinity II UPLC (Agilent Technologies, Santa Clara, CA, USA) was employed, which consists of a binary pump (G7120A), a multi-sampler (G7167B), an in-line degasser, and a variable-wavelength detector (VWD G7114B) adjusted to 214 nm. The hydrolysates were previously filtered using 0.22 µm polyvinylidene fluoride (PVDF) filters. Then, 1 µL of the samples was injected onto a Poroshell 120 EC-C18 column (2.1 × 100 mm i.d., 2.7 micron, Agilent, Santa Clara, CA, USA), which was operated at 23 °C with a flow rate of 500 µL/min. A linear gradient consisting of solvent A (LC-MS-grade water with 0.1% formic acid) and solvent B (LC-MS-grade ACN with 0.1% formic acid) was applied, with solvent B going from 1% to 65% in 25 min, ramping up to 95% and holding for 5 min, then, going back to the initial conditions.

The identification of peptides was performed via LC-MS/MS in an Agilent 6560 ion mobility quadrupole time-of-flight (IM-Q-TOF) mass spectrometer (Agilent, Santa Clara, CA, USA). Signals were acquired in positive mode at an extended dynamic range, 2 GHz, 3200 *m*/*z*, with a scan range between 100 and 3200 *m*/*z*. Nitrogen was used both as drying gas (13.0 L/min and 150 °C) and as nebulizer gas (30 psig). The capillary, nozzle, and fragmentor voltages were set at 3500 V, 300 V, and 400 V, respectively. The equipment was calibrated using an ESI-L low-concentration tuning mix (G1969-85000, Agilent Technologies, Santa Clara, CA, USA). Finally, the data acquisition and analysis were carried out through the Agilent Mass Hunter Software package (LC/MS Data Acquisition, Version B.08.00 and Qualitative Analysis for IM-MS, Version B.07.00 Service Pack 2 with BioConfirm Software). Complementarily, the possible peptide sequences of unidentified peptides were examined using the FindPept database (http://ca.expasy.org/tools/findpept.html, accessed on 15 February 2022); their molecular masses were employed and compared with the sequences of alpha-chain (α-globin) and beta-chain (β-globin) bovine or porcine hemoglobin.

Additionally, peptide quantification was performed. The molar concentration of peptides was determined by Equation (2), which was based on the UV peak areas recovered from RP-UPLC chromatograms at 214 nm [54].

(2) $$C_{\mathrm{pep}}=1\times 10^{6}\left(\frac{A_{214}}{\varepsilon_{214}{\;L\;V}_{\mathrm{inj}}{\;K}_{\mathrm{cell}}}\right)f$$

where C_pep_ (µM) is the concentration of peptides, A_214_ (AU min) is the RP-UPLC peak area of the absorbance at 214 nm, L (cm) is the path length of the UV cell (1 cm), V_inj_ (μL) is the injection volume (1 μL), *f* is the flow rate (500 μL/min), *K*_cell_ is the cell constant (which was previously determined to be 0.06 via calibration using pure peptide solutions of neokyotorphin (TSKYR) at known concentrations, assessed using the same parameter of analysis) [17], and *ε*_214_ (1/M cm) is the molar extinction coefficient at 214 nm, determined for each peptide according to Kuipers and Gruppen [55]. Co-elution of peptides was observed in some RP-UPLC peaks. Thus, C_pep_ was corrected by dividing the peak area by the number of peptides observed in the same RP-UPLC peak. In this case, an equal contribution to absorbance of the peptides was assumed.

### 2.6. Determination of the Protein Content of the Hydrolysates

The protein content of the hydrolysates was determined to produce hydrolysate solutions at known concentrations for the antimicrobial and antioxidant assays. Treated and untreated hydrolysates that produced up to 3 h of enzymatic digestion and adjusted to pH 3, 7, and 10 were freeze-dried and their protein content was estimated using the Dumas method [56] through a rapid Micro N cube (Elementar, Langenselbold, Germany), using 6.25 as a nitrogen-to-protein conversion factor.

### 2.7. Determination of the Antimicrobial Activity

In order to evaluate the impact of PEFs and pH on the antimicrobial activity of Hb-B and Hb-P hydrolysates, the antibacterial, antifungal, and anti-yeast activities of treated and untreated hydrolysates were studied. For this purpose, 4 target strains (Metabiolac collection, Université Laval) were assessed. *Listeria ivanovii* HP B28 (Gram^+^ bacteria) and *Escherichia coli* MP 4100 (Gram^−^ bacteria) were assessed for antibacterial activity. *Escherichia coli* was chosen since it is a common foodborne pathogen, while *Listeria ivanovii* was selected because it is a non-pathogenic strain, which can give an idea about the prevention of listeriosis (an issue for some foods) [17]. Antifungal and anti-yeast activities were evaluated for *Mucor racemosus* LMA-722 (filamentous fungal strain) and *Rhodotorula mucilaginosa* 27,173 (yeast strain), respectively. These strains were chosen as references for common mold and yeast involved in food spoilage [57]. The antimicrobial tests were carried out in two stages. First, growth inhibition was evaluated using an agar-well diffusion test. Then, the minimum inhibitory concentration (MIC) of hydrolysates was determined, as well as their minimum bactericidal concentrations (MBC) or minimum fungicidal concentrations (MFC) to establish their antimicrobial mechanism of action (-cidal or -static effect).

#### 2.7.1. Agar-Well Diffusion Assay

The agar-well diffusion assay was performed as described in detail in our previous study [17]. Briefly, the culture media were inoculated with the target strains. In the case of bacterial strains, 250 µL of an 18 h subculture of each strain (~1 × 10^6^ CFU/mL) was added to 25 mL of tryptic soy agar at 45 °C. For mold and yeast strains, potato dextrose agar (45 °C) inoculated with 250 µL of spore solution (mold) or vegetative cells (yeast) at a concentration of ~1 × 10^6^ cells/mL was used. Once the media were solidified in sterile Petri dishes, the wells were made using a sterile pipette. Then, 80 µL of each hydrolysate was deposited at a concentration of 40 mg/mL, which was established based on our previous study [17]. Only one concentration was selected, considering that this study aimed to screen the effect of PEF treatment and final pH on antibacterial activity. A negative control (sterile water) was used, as well as a positive control of ampicillin for bacterial strains (256 µg/mL) or natamycin (16.7 µg/mL) for mold and yeast strains. The petri dishes were incubated for 24 h for bacteria (37 °C for *E. coli* and 30 °C for *L. ivanovii*) and 48 h at 25 °C for *M. racemosus* and *R. mucilaginosa*. Finally, the diameter (mm) of the growth inhibition halo was measured.

#### 2.7.2. Determination of MIC, MBC, and MFC

The MIC, MBC, and MFC were also established according to our previous study via liquid growth inhibition assay in a 96-well polystyrene micro-plate (Becton Dickinson Labware, Sparks, MD, USA) [17]. The wells were loaded with a twofold serial dilution of each hydrolysate (40 mg/mL) in TSB for bacteria strains or with PDB for fungal and yeast strains. They were also inoculated with 50 μL of the target strains with a concentration between 0.5 and 1.0 × 10^6^ CFU/mL (bacteria) or cells/mL (mold and yeast). The micro-plates with *L. ivanovii* (30 °C) and *E. coli* (37 °C) were incubated for 24 h, whereas micro-plates with *M. racemosus* and *R. mucilaginosa* were incubated at 25 °C for 48 h. After incubation, the absorbance (595 nm) was measured using an Infinite F200 PRO photometer (Tecan US Inc., Durham, NC, USA). The MIC was determined considering the lowest concentration of the hydrolysate, showing complete inhibition of the growth of the target strain, which showed an optical density equal to the uninoculated culture medium (tryptic soy broth or potato dextrose broth). The solution of the wells without growth after incubation was used to estimate the MBC and MFC. The solutions (10 µL) were spread onto a tryptic soy agar Petri dish or DRBC agar and incubated under optimal temperature and time for each strain. The lowest concentration without microbial growth indicated the MBC or MFC. The mechanism of action of hydrolysates with an MBC/MIC ratio ≤ 4 was described as bactericidal, while a bacteriostatic effect was attributed to an MBC/MIC ratio > 4. The fungicidal (MFC/MIC ≤ 4) and fungistatic (MFC/MIC > 4) effects were also defined under the same criteria [58]. The MIC, MBC, and MFC values for ampicillin and natamycin (positive controls) were also determined.

### 2.8. Determination of Antioxidant Activity

#### 2.8.1. DPPH Free-Radical-Scavenging Capacity

The DPPH (1,1-diphenyl-2-picrylhydrazyl)-radical-scavenging capacity of bovine and porcine hydrolysates was evaluated according to Tremblay et al. [59] with some modifications. An aliquot (50 μL) of each sample prepared in ultrapure water at different concentrations (10, 5, 2.5, and 1.25 mg/mL) was mixed with 250 μL of 90 μM DPPH dissolved in 95% ethanol. The samples were incubated for 1 h at 30 °C in the dark, and the absorbance was recorded at 517 nm using a microplate spectrophotometer (xMark, Bio-Rad, Hercules, CA, USA). Ethanol (95%) was the control, and the procedure was the same as mentioned above. DPPH-radical-scavenging activity was calculated using Equation (3).

(3) $$\mathrm{DPPH}\;\mathrm{radical}\;\mathrm{scavenging}\;\mathrm{activity}\;\left(\%\right)=\frac{A_{\mathrm{control}}-A_{\mathrm{sample}}}{A_{\mathrm{control}}}\times 100$$

where A_control_ is the absorbance of the control reaction (containing all reagents except the sample) and A_sample_ is the absorbance of samples (with the DPPH solution).

#### 2.8.2. Oxygen-Radical Antioxidant Capacity (ORAC) Assay

The antioxidant activity of hydrolysates was also measured using the oxygen-radical absorbance capacity (ORAC) assay according to Tremblay et al. [59]. All reagents and samples were prepared in a sodium phosphate buffer (75 mM, pH 7.4). Different concentrations of both treated and untreated hydrolysates (0.313, 0.156, 0.078, and 0.039 mg/mL) were tested. Briefly, 25 µL of diluted sample and 150 µL of 0.4 µM fluorescein were added into the internal wells of a black 96-well microplate (Black U-Shape, Greiner Bio-One, Frickenhausen, Germany) and immediately incubated at 37 °C for 30 min in a Synergy H1 Hybrid Multi-Mode Microplate Reader (BioTek, Winooski, VT, USA). Then, 50 μL of 2,2′-azobis(2-methylpropionamidine) dihydrochloride (AAPH) solution (150 mM) was added rapidly and the fluorescence was recorded at 485 nm (excitation) and 538 nm (emission) at 37 °C every 1 min for 90 min. The net area difference under the curves of fluorescence decay for the blank and each sample was determined to enable the calculation of the ORAC values. Trolox was used as a standard reference compound (6.25–200 µM; R^2^ = 0.997) and the results were expressed as µM Trolox Equivalent (µM TEq).

### 2.9. Statistical Analysis

All experiments were carried out in triplicate and the values are reported as mean ± standard deviation. ANOVA (*p* < 0.05), followed by multiple range comparison (Tukey tests), was used for the analysis of the DH, antimicrobial, and antioxidant results using SigmaPlot Version 12.0 (Systat Software, Inc. San Jose, CA, USA). Heat maps of the peptide population results were created using RStudio Version 1.4.

## 3. Results and Discussion

### 3.1. Degree of Hydrolysis of Hydrolysates

**Figure 3:**
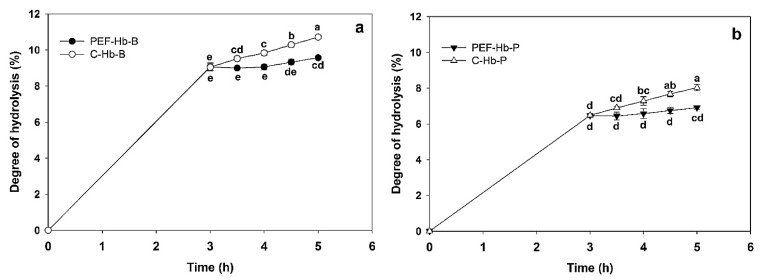
Evolution of degree of hydrolysis during enzymatic hydrolysis with pepsin of bovine (**a**) and porcine (**b**) hemoglobin untreated (C-Hb, control) and treated with PEFs after 3 h of hydrolysis. Different letters indicate a significant difference both between treatments and over time (Tukey test at 5%).

The DH of hemoglobin hydrolysates was studied as a control variable to verify the inactivation of pepsin after PEF treatment. Figure 3 depicts the DH for PEF-treated and untreated pepsin hydrolysates produced from Hb-B and Hb-P. It can be observed that the treated and untreated samples presented similar values at 3 h of hydrolysis. The hydrolysates achieved values of 9.05 ± 0.19% and 6.47 ± 0.06% for Hb-B and Hb-P, respectively, indicating that the hydrolysates were properly prepared. Those values were slightly lower than the ones reported by Sanchez-Reinoso et al. [17], who evaluated the hydrolysis of bovine (DH = 9.54 ± 0.35%) and porcine hemoglobin (8.4 ± 0.2%) with pepsin under the same conditions of pH (pH 3), temperature (37 °C), and hydrolysis time (3 h) but with a different hemoglobin concentration (1% *w*/*w*) than in the present study (0.5% *w*/*w*). Differences in DH between Hb-B and Hb-P hydrolysates can be explained by the difference in the sequences of Hb-B and Hb-P. The alpha and beta chains of bovine and porcine hemoglobin were only 86.5 and 82.6% similar, respectively [17]. For this reason, some cleavage sites of pepsin differed. Indeed, Zouari et al. [18] reported that according to in silico predictions, Hb-B had 3 additional pepsin cleavage sites on the alpha chain and 10 different cleavage sites on the beta chain in comparison to Hb-P. As a consequence, pepsin could have higher specificity on Hb-B and, consequently, a higher DH, which represents a greater number of free amino groups released by the cleavage of peptide bonds during the peptic hydrolysis of hemoglobin.

In addition, both bovine and porcine hydrolysates showed a similar evolution of DH with time. The DH for control samples (untreated) significantly increased as the treatment time increased due to the enzymatic action (*p* < 0.05), reaching values of 10.71 ± 0.05% for Hb-B and 8.03 ± 0.17% for Hb-P after 5 h of hydrolysis. Conversely, hydrolysates treated with PEFs after 3 h of hydrolysis showed constant behavior in their DH. This indicated that PEFs had an impact on the enzymatic activity of pepsin, hampering its interactions with hemoglobin.

Comparing the DH evolution of samples treated with PEFs through time, no statistical differences were found for bovine hydrolysates until 4.5 h, but a slight increase was observed after 5 h (Figure 3a). This slight increase in DH could suggest possible reactivation of pepsin, maybe due to posterior refolding of the enzyme. Moreover, the DH of porcine hydrolysates (Figure 3b) did not change significantly during the different times tested post-PEF treatment. Consequently, the DH analysis suggested that treatment of Hb-B and Hb-P hydrolysates generated an impact on pepsin activity, mitigating its enzymatic action such that the proportion of peptides in the crude hydrolysates varied to a lesser extent than the untreated hydrolysates. This may be associated with the alteration of the tertiary and secondary structures of pepsin generated by PEF treatment. It has been described that PEFs with a field strength between 20 and 40 kV/cm lead to the loss of the β-sheet structure constituting pepsin and, consequently, its enzymatic activity, which decreases with the enhancement of electric-field strength [42,43,60]. However, the severity of the PEF conditions tested seems not to be enough to completely denature pepsin since a slight increase in DH may lead to partial restoration of its three-dimensional structure (renaturation) and enzymatic activity. Indeed, such a phenomenon was reported by Salvia-Trujillo et al. [61] for polygalacturonase after its inactivation by PEFs in fruit juice–milk beverages.

### 3.2. RP-UPLC-MS/MS Characterization of Hydrolysates

#### Peptide Profiles of Hydrolysates

The behavior of the peptide population in the control and PEF-pretreated hydrolysates was studied as a complementary method of DH analysis to verify pepsin inactivation. A post-PEF-treatment variation in the peptide population might indicate that pepsin continued its enzymatic action, whereas a constant peptide population might indicate a loss of enzymatic action on the substrate. For this purpose, the peptide concentration of treated and untreated hydrolysates of Hb-B and Hb-P at 3, 4, and 5 h of hydrolysis was determined and compared. In general, the bovine hydrolysates presented a higher number of peptides (144 peptides) than the porcine hydrolysates (127 peptides). This is in accordance with the differences observed for DH since a higher number of peptides released in Hb-B hydrolysates lead to a higher DH value compared to Hb-P hydrolysates. Similar to DH behavior, this variation between hemoglobin sources may be due to their differences in sequences and pepsin cleavage sites [18].

**Figure 4:**
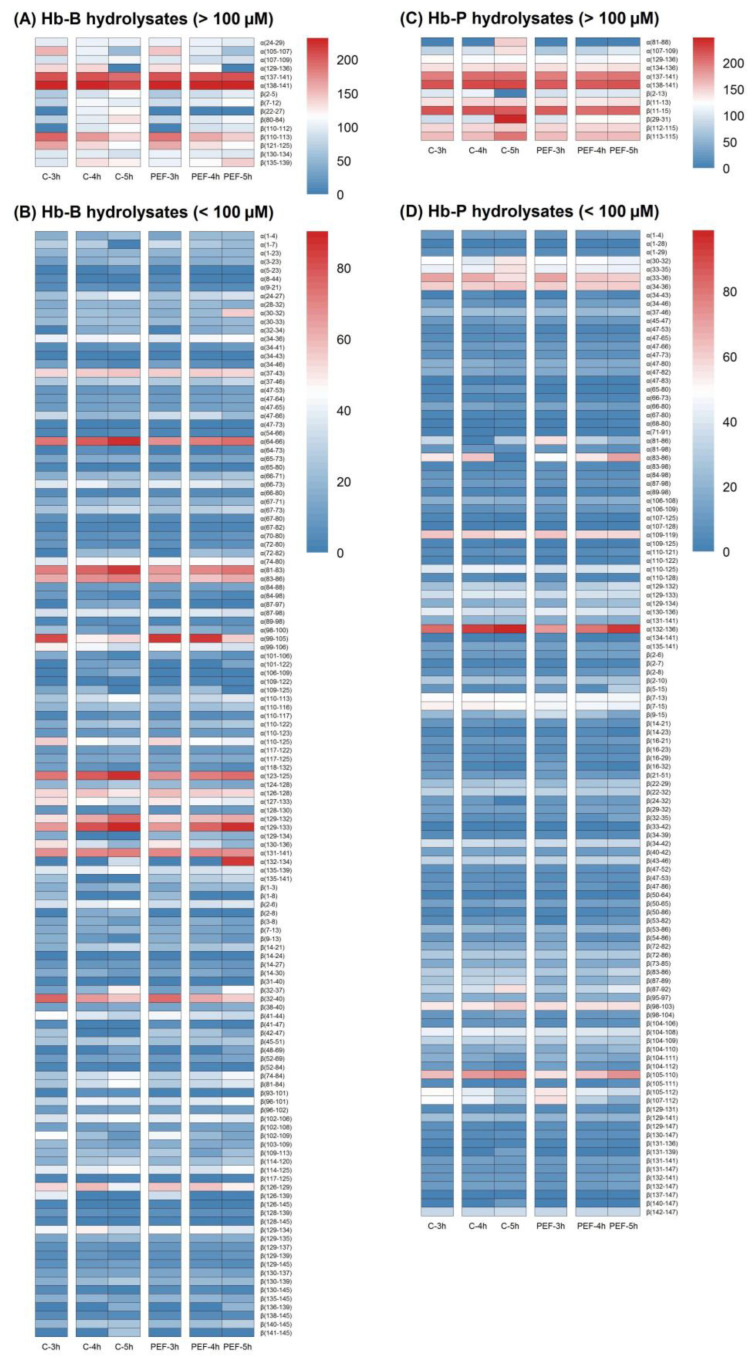
Heat map based on peptide concentration (µM) of identified peptides in untreated (C, control) and PEF-treated Hb-B (**A**,**B**) and Hb-P (**C**,**D**) hydrolysates. Identified compounds are labeled by their location in the sequences of alpha chain or beta chain. Treatments and peptides are visualized in each column and row, respectively. Values in the diagram were calculated from the average of 3 replicate samples.

Figure 4 depicts the heat maps of the peptide concentration of the hydrolysates produced from Hb-B and Hb-P. In general, high similarity in the peptide population of PEF-treated and untreated hydrolysates at different times was observed in both Hb-B and Hb-P hydrolysates. This can be explained by the hydrolysis time considered for the present study since after 3 h of peptic hydrolysis, a plateau trend in hydrolysis has almost been reached. This behavior has also been observed by Sanchez-Reinoso et al. [17] and it might indicate that the substrate (Hb-B or Hb-P) has been almost entirely consumed, which may lead to slowing down of enzymatic hydrolysis and, consequently, to lower peptide diversity. Nevertheless, some differences between treated and untreated hydrolysates were observed when comparing samples over time. Both Hb-B and Hb-P hydrolysates treated with PEFs presented peptides that did not change their concentration throughout time or that varied in a smaller proportion than the untreated hydrolysates. Peptides with a concentration > 10 µM such as α(1–7) (28 µM), α(109–125) (13 µM), α(129–134) (15 µM), α(129–136) (136 µM), α(135–141) (23 µM), β(1–8) (20 µM), β(42–47) (24 µM), and β(126–139) (38 µM), as well as peptides with a low concentration such as α(8–44) (5 µM), α(47–73) (3 µM), α(65–80) (2 µM), β(41–47) (4 µM), β(52–84) (2 µM), β(126–145) (2 µM), and β(128–145) (2 µM) were produced during the first 3 h of hydrolysis in untreated Hb-B hydrolysates; however, these peptides disappeared after 5 h of the process (Figure 4A,B), indicating a significant difference through time according to the Tukey test (*p* < 0.05). The enzymatic mechanism developed during hydrolysis could be the reason for this behavior. In the case of the hydrolysis of bovine hemoglobin with pepsin, the mechanism of action at pH < 3.5 has been described as the Zipper type, in which pepsin rapidly leads to the formation of intermediate peptides, which, in turn, are hydrolyzed into smaller peptides as hydrolysis progresses [62]. When comparing the behavior of the above-mentioned peptides in samples treated with PEFs, α(8–44) α(47–73), α(65–80), α(109–125), α(129–136), β(1–8), β(41–47), β(52–84), β(126–139), β(126–145), and β(128–145) also disappeared after 5 h. Nonetheless, certain peptides with higher initial concentrations (>10 µM) were still present at relatively similar concentrations after 5 h under optimal pH and temperature conditions (pH 3 at 37 °C) for pepsin such as α(1–7) (21 µM), α(129–134) (17 µM), α(135–141) (31 µM), and β(42–47) (14 µM), whereas the peptide β(41–47) was retained until 4 h of hydrolysis, even though its initial concentration was very low (3 µM). Although several of these peptides were also degraded as a function of time in PEF-treated hydrolysates, the presence of the above-mentioned peptides suggests that the enzymatic activity of pepsin was weakened. This hypothesis is also supported by the formation of peptides after 3 h of enzymatic hydrolysis in untreated hydrolysates that were not observed in PEF-treated samples. In this case, the peptides α(32–34) (15 µM), α(64–73) (6 µM), α(72–82) (20 µM), α(87–97) (14 µM), α(101–122) (11 µM), α(106–109) (11 µM), β(2–8) (15 µM), β(22–27) (106 µM), β(93–101) (6 µM), and β(110–112) (95 µM) were formed from 4 h of hydrolysis, whereas the peptides α(5–23) (7 µM), α(132–134) (33 µM), β(14–24) (2 µM), β(48–69) (12 µM), β(136–139) (17 µM), and β(141–145) (25 µM) were observed only after 5 h. Among the latter-mentioned peptides, the peptides α(5–23), α(87–97), α(106–109), β(2–8), β(14–24), β(22–27), β(93–101), and β(141–145) present at concentrations ranging from 2 to 106 µM in untreated hydrolysates were not observed in the PEF-treated hydrolysates; this was probably due to the decrease in pepsin activity resulting from PEFs. In addition, the PEF-treated hydrolysates also showed other peptides that varied to a smaller extent in concentration over time than the untreated samples. Among them, α(67–71), α(89–98), α(101–106), β(80–84), β(102–109), and β(110–112) varied at least 0.5 times less in concentration than the untreated hydrolysates after 5 h of hydrolysis. These results would indicate that PEFs had an impact on the enzymatic activity of pepsin, suggesting that pepsin was at least partially inactivated.

Similarly, hydrolysates produced from Hb-P and treated with PEFs showed fewer changes in the peptide population than untreated hydrolysates (Figure 4C,D). As with Hb-B hydrolysates, some peptides were observed to remain after 5 h in PEF-treated Hb-P hydrolysates, but not in untreated samples. A significant decrease (or absence) was observed for the peptides α(83–86), α(107–125), α(110–128), β(2–13), and β(24–32) in untreated Hb-P hydrolysates (*p* < 0.05), whereas the same peptides were maintained during the 5 h in hydrolysates up to concentrations of 103 µM for β(2–13), 69 µM for α(83–86), 10 µM for β(24–32), 2 µM for α(107–125), and 2 µM for α(110–128). Additionally, conventional hydrolysis of Hb-P during 5 h of digestion resulted in the formation of the peptides α(81–88), β(131–139), and β(140–147) up to concentrations of 149, 14, and 11 µM, respectively. These peptides were not observed at 3 h of hydrolysis, which would indicate a significant increase in their concentration over time (*p* < 0.05). In contrast, these peptides were not generated in PEF-treated hydrolysates, likely due to the positive impact of the treatment on pepsin inactivation. Lastly, peptides that varied in a smaller proportion in the treated hydrolysates than in the untreated ones at 5 h were also identified, such as α(33–35), α(65–80), α(107–109), β(29–31), β(87–92), and β(113–115). These peptides showed at least a 0.5-fold lower variation than untreated samples in their concentration.

According to these results, it can be mentioned that PEF treatment of Hb-B and Hb-P hydrolysates had an impact on pepsin activity by mitigating its enzymatic action such that the proportion of peptides in the crude hydrolysates varied to a lesser extent than the untreated hydrolysates, which is consistent with the results observed in the DH. Nonetheless, variations in the peptide population of treated samples would indicate residual pepsin activity. These changes in the peptide population of the PEF-treated hydrolysates were not evidenced in the DH behavior (Figure 3), where a clearer variation was observed in untreated samples. This can be explained by the method used in the determination of DH (OPA test), which considered both the peptides and free amino acids of the hydrolysates. In contrast, some di-peptides and free amino acids were not considered in the analysis of the peptide population due to the sensitivity limit of the MS/MS method. Another explanation may be that some peptides can disappear (or their concentration can decrease), while others can appear (or their concentration can increase) in RP-UPLC-MS/MS spectra, such as the peptides α(129–136), β(141–145), and β(22–27) for Hb-B hydrolysates and the peptides α(81–88), α(132–136), and β(2–13) for Hb-P hydrolysates; on the other hand, DH indicated only a release of free amino groups, which prevents us from understanding the change in a peptide population. This also supports the necessity of performing RP-UPLC-MS/MS analysis, including the estimation of peptide concentration, to prove enzyme inactivation.

### 3.3. Determination of Antimicrobial Activity

#### 3.3.1. Antibacterial Activity

The zone of inhibition results against *E. coli* and *L. ivanovii* for the PEF-treated and untreated hydrolysates of Hb-B and Hb-P at different pH values are presented in Table 1. As observed, Hb-B and Hb-P hydrolysates did not show any antimicrobial activity against the *E. coli* strain. This is consistent with our previously reported study under similar enzymatic hydrolysis conditions (pH 3, 3 h, E/S ratio of 1:11 mol/mol) [17], where no antimicrobial activity was observed for Hb-B or Hb-P hydrolysates. Conversely to other studies, peptides produced from Hb-B have an antibacterial effect against *E. coli* [4,8,9,10,11,12,15]. The absence of inhibition could be explained by the absence of several intermediate peptides formed at the beginning of enzymatic hydrolysis (up to a DH of 3%), such as α(107–133), α(107–136) [14,15], α(107–141), α(133–141) [10,14,15], β(1–30) [14], β(114–145), and β(121–145) [9,14], which could have been hydrolyzed into minor peptides (e.g., α(107–109), α(110–113), α(117–122), α(135–139), β(1–3), β(1–8), β(2–5), β(114–120), β(121–125), etc.). Moreover, they may contribute to strong antimicrobial activity against this Gram^−^ bacteria. These results would also indicate that PEF treatment also had no effect on the enhancement of antimicrobial activity against *E. coli*.

Regarding *L. ivanovii*, all the hydrolysates showed an antibacterial effect against this Gram^+^ bacteria. The Hb-P hydrolysates showed larger diameters of growth inhibition (7.7–10.7 mm) in comparison to the Hb-B hydrolysates, which ranged from 7.3 to 8.3 mm (Table 1). According to multiple comparisons via Tukey tests, there were no significant differences between PEF-treated and untreated hydrolysates for this strain, but the inhibition zone diameters of all the hydrolysates were significantly smaller than those of the ampicillin control (*p* < 0.05). In addition, the pH value of hydrolysates also showed no significant effect on antibacterial activity. The impact of PEFs and pH on the MIC, MBC, and MBC/MIC ratio of hydrolysates against *L. ivanovii* was also studied (Table 2). The MIC confirmed the zone of inhibition results since both PEF treatment and pH did not have a significant impact on MIC. The hydrolysates of Hb-P presented lower MIC (2.5 mg/mL) than the Hb-B hydrolysates (5.0 mg/mL), which indicates that Hb-P has stronger antibacterial activity against *L. ivanovii* (*p* < 0.05). Similar behavior was observed by Sanchez-Reinoso et al. [17], who reported that peptic hydrolysates from Hb-P produced under similar enzymatic hydrolysis conditions (pH 3 at 37 °C for 3 h) had stronger antibacterial activity (MIC = 0.62 mg/mL) than Hb-B hydrolysates (MIC = 1.25 mg/mL) against the same strain, which could be explained by the differences in the Hb-B and Hb-P sequences. Regarding differences in MIC versus the previous study, this may be due to the lower initial hemoglobin concentration (0.5% *w*/*w*) and production volume (1 L) used in this study, which might have negatively affected the pepsin activity. Evidence supporting this hypothesis is the lower DH obtained in the present study (9.05% for Hb-B and 6.47% for Hb-P) compared to our previous study (9.54% for Hb-B and 8.41% for Hb-P). Furthermore, MBC for *L. ivanovii* was also not affected by the PEF treatment, but hydrolysates at pH 3 showed a lower MBC value than those at pH 7 and 10 (MBC > 20 mg/mL). This indicates that an acidic pH in the samples had an impact on the MBC, fostering stronger bactericidal activity. These MIC values were lower, and hence, had stronger antibacterial activity, in comparison with hydrolysates produced from other native proteins such as WPI using pepsin (MIC = 75 mg/mL) for the inhibition of *L. ivanovii* [63], or tryptic hydrolysates from WPI (MIC = 20 mg/mL) for the same target strain [64]. Additionally, even though MIC was stronger for Hb-P hydrolysates, the type of hemoglobin did not show a difference in the MBC. This fact indicates that the source of hemoglobin may affect the inhibition of growth but not the bactericidal effect against the target strain. Eventually, the pH value also impacted the type of antimicrobial action since MBC/MIC values ≤ 4 were obtained for pH 3, indicating bactericidal action on *L. ivanovii*, whereas the hydrolysates at pH 7 and pH 10 obtained MBC/MIC values > 4, which suggests that hydrolysate acted as a bacteriostatic agent [65]. According to these results, the PEF treatments had neither a positive nor a negative impact on the antibacterial activity of the hydrolysates. On the contrary, pH 3 could favor the bactericidal action of hemoglobin hydrolysates against Gram^+^ strains. This could be related to the protonation of histidine in antimicrobial peptides at an acidic pH, which can promote electrostatic interactions with negatively charged cell wall components that are Gram^+^ such as wall- and lipoteichoic acids [25]. Several antimicrobial peptides containing histidine have been previously reported in Hb-B and Hb-P hydrolysates [17]. Finally, it is important to mention that native hemoglobin controls were included at different pH values. In this case, no native hemoglobin solution showed antibacterial activity.

##### 3.3.2. Antifungal and Anti-Yeast Activity

The antifungal and anti-yeast activities are also presented in Table 1 and Table 2. Growth inhibition against *M. racemosus* was only observed for hydrolysates at pH 7 and 10 with a stronger effect at alkaline pH (*p* < 0.05), in which the diameter of growth inhibition varied from 9.7 to 12.7 mm for Hb-B hydrolysates and 10.3 to 15.3 mm for Hb-P hydrolysates. The PEF-treated hydrolysates were not significantly different from the untreated samples. The observed MIC and MFC values had the same tendency for PEF-treatment effects (Table 2), where the treated and untreated hydrolysates were similar between them. The MIC and MFC values obtained for Hb-B hydrolysates were, respectively, 5 mg/mL at pH 7 and >20 mg/mL at pH 10, which confirmed that antifungal activity changed with the pH variation observed in the zone of growth inhibition. These results for MIC differ from those reported by Abou-Diab et al. [23] in the conventional hydrolysis of Hb-B with pepsin at pH 3 for 3 h at 30 °C, whereby they found an MIC of 10 mg/mL against *M. racemosus*. They were also different from the results reported by Sanchez-Reinoso et al. [17], who observed no antifungal activity against *M. racemosus* in Hb-B hydrolysates. This could also be associated with variation in the DH mentioned above and, consequently, a variation in the peptide population. This would suggest that a lower DH could lead to stronger antifungal activity. Meanwhile, the MIC for Hb-P hydrolysates was not different between pH 7 and 10, values that agree with the MIC (1.25 ± 0.02) found by the same authors in peptic hydrolysates of Hb-P at pH 3, at 37 °C for 3 h. Nonetheless, the MFC was lower (1.25 ± 0.00) than the one reported in the same study (2.49 ± 0.00). Finally, note that both Hb-B and Hb-P hydrolysates had an MFC/MIC ratio of 1. Hence, these hydrolysates act as fungicidal agents (MFC/MIC ratio < 4) [58] on the target strain at a concentration ≥ 1.25 mg/mL.

Furthermore, all the hydrolysates showed an anti-yeast effect (Table 1). It can easily be noticed that *R. mucilaginosa* had a higher sensibility to Hb-P hydrolysates compared to Hb-B hydrolysates. Additionally, a significant effect of both PEF treatment and pH value was observed on the diameter of the growth inhibition zones. Hb-B hydrolysates at pH 3 and that were PEF-treated were significantly lower (9.7 ± 1.2 mm) than their control at pH 3 (15.3 ± 0.6 mm) according to Tukey’s test (*p* < 0.05). The same significant difference was found in Hb-P hydrolysates at pH 3 treated with PEFs (17.3 ± 0.6 mm) and their respective control at pH 3 (21.0 ± 1.0 mm). However, these differences were not observed between bovine and porcine hydrolysates at pH 7 and 10. These results are similar to the ones reported previously for the peptic hydrolysates of Hb-B (17.7 ± 0.7 mm) and Hb-P (21.3 ± 4.2 mm) against *R. mucilaginosa* at pH 10 [17].

As can be observed in Table 2, a clear tendency toward a decrease in MIC values for *R. mucilaginosa* as the pH increased was observed in Hb-B hydrolysates (*p* < 0.05). The MICs of Hb-B hydrolysates treated with PEFs were 10 mg/L for pH 3, 1.25 mg/L for pH 7, and 0.63 mg/L for pH 10, whereas their controls at pH 3, 7, and 10 were 2.50, 1.25, and 0.63 mg/L, respectively. Those values make it clear that PEF treatment led to lower anti-yeast activity (8 times) against *R. mucilaginosa* when the pH value of hydrolysates was adjusted to 3 in comparison to the untreated hydrolysates. In the same way, acid aqueous media led to lower anti-yeast activity against the target strain. In contrast to what was observed for Hb-B hydrolysates, no clear trend was observed for the effect of pH in Hb-P hydrolysates. Only hydrolysates treated at pH 3 and untreated at pH 10 were different from the other treatments. However, the PEF-treated porcine hydrolysates also exhibited lower anti-yeast activity (8 times) at pH 3 than their untreated control at pH 3. Regarding the MFC, PEFs had some effect on the bactericidal effect of Hb-B hydrolysates. Treated Hb-B hydrolysates at pH 3 (20.00 ± 0.00) and 10 (1.25 ± 0.00 g) were significantly higher than untreated hydrolysates at pH 3 (5.00 ± 0.00) and 10 (0.63 ± 0.00). The same significant difference was found in Hb-P hydrolysates at pH 3 (10.00 ± 0.00) in comparison to the control at the same pH value (0.63 ± 0.00). Finally, it is important to mention that even though differences were found between treatments, all the hydrolysates presented values of MBC/MIC ratio lower than 4. This suggests that the hydrolysates had fungicidal action against *R. mucilaginosa* in an equal or higher concentration than their MICs [65]. These results agree with the ones reported by Sanchez-Reinoso et al. [17], who discovered a fungicidal effect of Hb-B and Hb-P hydrolysates against yeast strain (*R. mucilaginosa*). According to these results, we can mention that the PEF and pH values of hydrolysates could generate some changes in the anti-yeast activity of bovine and porcine hemoglobin hydrolysates, particularly when the treated hydrolysates are kept at pH 3.

To summarize, it can be mentioned that PEF treatments had no significant impact on the antibacterial and antifungal activities of Hb-B and Hb-P hydrolysates. However, some negative impacts on anti-yeast activity were observed, particularly when the hydrolysates were in acidic media (pH 3), which showed much higher MIC and MFC compared to neutral and alkaline media. Likewise, the important role of pH of the hydrolysates in their antimicrobial activity was elucidated, which gives a particular idea of the possible products in which they could be applied. Another aspect to highlight is that, unlike the antibacterial and antifungal activities, anti-yeast activity was observed in native hemoglobin. The native Hb-B solutions at pH 3 presented anti-yeast activity against *R. mucilaginosa*, while the native Hb-P solutions presented anti-yeast activity against the same target strain at pH 3 and to a lesser extent at pH 10 (Table 1 and Table 2). Variation in hemoglobin pH can lead to the protonation (acidic pH) or deprotonation (basic pH) of histidine residues, which are part of the hemoglobin protein; this can result in major rearrangements in the tertiary or quaternary structure of the protein, promoting membrane interactions that lead to yeast inactivation [66]. These results would indicate, for the first time, a possible higher sensitivity of yeast strains to native hemoglobin solutions than bacteria and fungi.

### 3.4. Determination of Antioxidant Activity

#### 3.4.1. DPPH Free-Radical-Scavenging Capacity

**Figure 5:**
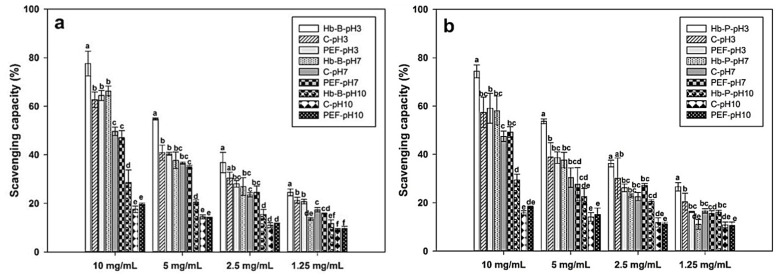
DPPH-scavenging activities of bovine (**a**) and porcine (**b**) hemoglobin hydrolysates that were untreated (C, control) and treated with PEFs at different concentrations. Hb-B and Hb-P indicate native bovine and porcine hemoglobin, respectively. Values with different lowercase letters within the same concentration are significantly different (Tukey test at 5%).

DPPH is one of the most-used antioxidant assays for the analysis of hemoglobin hydrolysates. This method is based on the scavenging of a stable free-radical DPPH• (2,2-diphenyl-1-picrylhydrazyl, λ_max_ = 517 nm) by antioxidants, which leaves a reduction reaction that decolorizes the DPPH molecule [67,68]. Figure 5 depicts the impact of PEF treatment, pH value, and concentration on the DPPH-radical-scavenging ability of Hb-B and Hb-P hydrolysates. In the same way, native hemoglobins at different pH levels were used as controls. As expected, the antioxidant activity of hydrolysates increased with increasing hydrolysate concentrations, which is directly associated with a higher content of antioxidants that can react with the DPPH radical. It is also noteworthy that the native hemoglobin samples showed higher scavenging capacity of the DPPH radical than their respective hydrolysates at the same pH value (*p* < 0.05). This behavior is contrary to that reported in other studies, where higher DPPH-scavenging capacity was found in hydrolyzed Hb-P than in its native form [13,22]. Disruption of the native hemoglobin structure by enzymatic hydrolysis perhaps leads to the exposure of active amino acids capable of reacting with oxidants. Perhaps an acidic pH can also induce such alterations, and therefore, native Hb-P showed stronger antioxidant activity at pH 3. In contrast, native Hb-P has exhibited higher hydroxyl-scavenging capacity and ferrous chelating ability than its hydrolysates produced via a non-enzymatic method [69].

Otherwise, it can be observed that there were no significant differences between untreated and PEF-treated samples in their DPPH-scavenging capacity. This suggested that the PEF treatment did not have an impact on the scavenging capacity against DPPH free radicals in Hb-B (Figure 5a) or Hb-P (Figure 5b) hydrolysates. These results differ from those obtained for peptide fractions of egg white and corn gluten proteins hydrolyzed with alcalase. Wang et al. [44] evaluated the impact of PEFs on the antioxidant activity of hydrolysates obtained using alcalase from egg white (pH 11, 50 °C for 3 h) and subsequent purification via ultrafiltration (1–10 kDa). In addition, they optimized the conditions of concentration, electric-field strength, and pulse frequency for PEF treatment, finding that peptides at 8 mg/mL, treated at 10 kV/cm and 2400 Hz, increased DPPH-inhibitory acti-vity by 28.44% (62.64 ± 0.98%). Similar results were subsequently found by Wang et al. [47], who reported a 32.1% increase in DPPH-inhibitory capacity in peptide fractions (10–30 kDa) of corn gluten hydrolysates (10 mg/mL) produced with alcalase (pH 10 at 60 °C) and treated at 15 kV/cm and 2000 Hz. This suggests that purification of the peptides prior to PEF treatment could favor their impact on the enhancement of antioxidant activity. Another explanation for the absence of enhancement of DPPH-inhibitory activity could be related to the conditions of PEF treatment. Considering the parameters applied for the enhancement of the antioxidant activity of peptides in continuous PEF systems, most studies have applied a higher pulse frequency (1600–3000 Hz) and lower flow rate (1.6–3.2 mL/min) [31] than those applied in this study (90 Hz and 100 mL/min). This favors a higher number of pulses applied to the samples, which could promote the development of the mechanism involved in the enhancement of the bioactivity of antioxidant peptides. This has been summarized by Zhang et al. [31] according to three main factors: (1) changes in their secondary structure, mainly in the loss of the α-helices structure; (2) alterations in the microenvironment of some H protons in aqueous solutions; and (3) changes in the spatial structure (e.g., approach trend between residues). Nevertheless, PEF treatment with a high frequency in continuous systems could lead to problems such as electric arc generation and a temperature increase inside the treatment chamber, whereas low flow rates cannot be applied for pepsin inactivation considering that PEF treatment must be applied to the whole solution as fast as possible. This problem can be solved in a real process by employing multiple treatment chambers in parallel, which could increase the retention time and the number of pulses applied with higher flow rates. Moreover, it is important to consider that requiring more electric-field pulses to develop these mechanisms could also involve higher energy consumption. For this reason, these variables must be balanced to achieve a more eco-efficient approach (the achievement of higher antioxidant activity with less energy consumption).

Moreover, the results clearly indicated that DPPH was affected by the pH value (*p* < 0.05). The strongest DPPH-scavenging activity was observed in acidic conditions (pH = 3), which decreased as the pH value increased. As mentioned above, pH variation can lead to protonation and deprotonation of the amino acid residues that constitute the hemoglobin-derived peptides [66], which can affect the antioxidant mechanisms developed during the DPPH•-scavenging reaction, such as hydrogen atom transfer (HAT), sequential electron–proton transfer (SEPT), proton-coupled electron transfer (PCET), and proton loss single-electron transfer (SPLET) [70]. In addition, a decrease in the hydrogen ion concentration leads to an increase in the reaction rate between DPPH and antioxidants [71]. The enzymatic hydrolysis time, and hence, the DH, can also both positively and negatively affect DPPH activity. Sun et al. [13] compared the effects of the type of protease (trypsin, Flavorzyme, papain, A.S.1398, alcalase, and pepsin) and hydrolysis time on the DPPH-scavenging of porcine hemoglobin. They observed that pepsin hydrolysates showed the strongest DPHH-scavenging capacity (67.0 ± 1.84%) at 1 h of hydrolysis, which decreased over time by ~17% at 8 h of hydrolysis since the antioxidant peptides could become hydrolyzed into lower-antioxidant ones. Conversely, Bah et al. [72] reported that the DPHH-scavenging capacity was increased over the hydrolysis time in hydrolysates of red blood cells from cattle and pigs using plant and fungal protease.

Another important aspect to mention is the similarity in DPPH-scavenging capacity between Hb-B and Hb-P hydrolysates, which would support the presence of antioxidant peptides independent of the hemoglobin source. In order to better understand the differences between the antioxidant peptide population between Hb-B and Hb-P hydrolysates, a computational approach was applied. The AnOxPePred database (https://services.healthtech.dtu.dk/service.php?AnOxPePred-1.0, accessed on 20 February 2022) was employed to predict the free-radical-scavenging (FRS) score, which varies from 0 (not antioxidant) to 1 (antioxidant) could describe peptides involved in the mechanisms of the DPPH•-scavenging reaction [73]. The predicted values of FRS score for Hb-B and Hb-P hydrolysates are presented in Table S1 and Table S2, respectively. The Hb-B and Hb-P hydrolysates presented an FRS of 0.248–0.574, which supports the presence of potential antioxidant peptides with free-radical-scavenging capacity. The peptides β(31–40), β(32–40), β(32–37), β(141–145), β(140–145), β(138–145), α(37–46), α(101–122), α(24–27), α(110–116), α(34–46), α(117–122), α(110–125), α(138–141), and β(135–145) presented the highest values of FRS among the peptide population of bovine hydrolysates. Peptides with identical sequences, and thus, equal FRS scores, but with different localization were found in Hb-P hydrolysates. Additionally, similar peptides with some differences in their sequences were also present. The differences and similarities of the peptides with higher FRS scores of Hb-B and Hb-P hydrolysates are compared in Table 3. As can be seen, the peptides β(33–42), β(34–42), β(34–39), α(37–46), α(138–141), β(98–104), β(98–103), α(84–98), and α(137–141) derived from Hb-P present the same sequence identity and FRS values as their Hb-B-derived counterparts. In contrast, the variations in some amino acids (K, G, A, H, D, N, S, and F) in the peptides β(142–147), β(140–147), α(34–46), α(110–125), β(137–147), α(47–53), β(132–147), α(66–80), α(65–80), and α(109–125) derived from Hb-P evidence their impact on the FRS scores. For example, the presence of amino acid K instead of amino acid R of the porcine peptide QKVVVAGVANALAHKYH decreases its potential free-radical-scavenging capacity from 0.427 (FRS score for QKVVAGVANALAHRYH) to 0.416. Likewise, this same amino acid (K) affects the FRS score of peptides derived from the same family, such as GVANALAHKYH, NALAHKYH, and LAHKYH. These differences could be studied in detail in future studies to elucidate the most appropriate source of antioxidant activity in hydrolysates from different hemoglobin sources.

#### 3.4.2. ORAC Test

**Figure 6:**
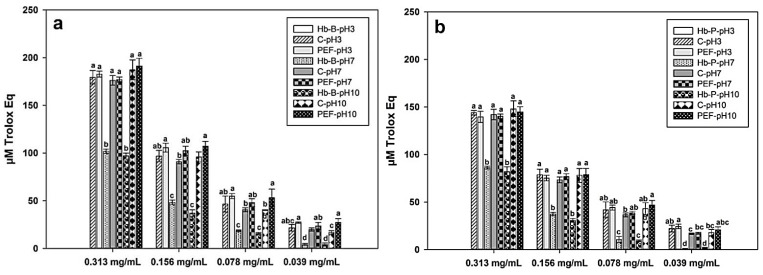
Antioxidant capacity of bovine (**a**) and porcine (**b**) hemoglobin hydrolysates obtained by the ORAC test (µM Trolox Eq) in untreated (C, control) and treated with PEFs at different concentrations. Hb-B and Hb-P indicate native bovine and porcine hemoglobin, respectively. Native Hb-B and Hb-P controls at pH 3 were not determined since they were not soluble in sodium phosphate buffer. Values with different lowercase letters within the same concentration are significantly different (Tukey test at 5%).

The ORAC test is a hydrogen atom transfer (HAT) assay based on the measurement of fluorescence quenching caused by peroxyl free radicals generated from 2,2′-azobis(2-methylpropionamidine) dihydrochloride (AAPH), which is inhibited in the presence of antioxidant compounds [74]. The results of the antioxidant activity of Hb-B and Hb-P hydrolysates determined by ORAC assay are presented in Figure 6. In general, both bovine and porcine hydrolysates presented higher ORAC values at the higher concentration of hydrolysate evaluated (0.313 mg/mL), which decreased with decreasing concentration by up to 80% at 0.039 mg/mL. Contrary to the results observed for DPPH-scavenging capacity, samples of native Hb-B and Hb-P at pH 7 and 10 had significantly lower Trolox Eq values than their peptic hydrolysates (*p* < 0.05). This suggests that hemoglobin hydrolysis releases peptides with oxygen-radical-scavenging capacity.

Bovine hydrolysates presented ORAC values ranging from 16.68 to 191.32 µM Trolox Eq, while porcine hydrolysates ranged from 16.52 to 148.11 µM Trolox Eq. Higher ORAC values were also observed by Bah et al. [72] in hydrolysates of bovine and porcine red blood cell fractions upon hydrolysis with the bromelain, papain, and FP400 (enzyme of fungal origin) enzymes. As with DPPH activity, PEF treatment did not show a significant difference in ORAC compared to the untreated samples for both bovine (Figure 6a) and porcine (Figure 6b) hydrolysates. This is contrary to the results reported by Zhang et al. [75], who improved the oxygen radical antioxidant capacity of the peptide QWFH from pine nut protein by 76.62% by applying PEFs of 15 kV/cm at 2400 Hz. This would support the hypothesis that PEF treatment under the conditions evaluated does not favor an improvement in the antioxidant activity of hemoglobin hydrolysates. Unlike DPPH-inhibitory activity, pH value showed no significant impact on ORAC. Only some significant differences in ORAC were observed (*p* < 0.05), particularly at lower concentrations of hydrolysates.

## 4. Conclusions

In the present study, the application of PEF treatments in the production of bioactive peptides via enzymatic hydrolysis was evaluated for the first time as an alternative for enzyme inactivation and subsequent strengthening of their bioactivity. Treatment with PEFs showed an impact on pepsin activity in bovine and porcine hemoglobin, maintaining a constant degree of hydrolysis and reducing variation in the peptide profile of the hydrolysates after PEF treatment. The study of antibacterial and antioxidant activities suggested that the hydrolysates did not improve their bioactivities under the conditions evaluated, nor were they negatively impacted, except for anti-yeast activity at pH 3. Moreover, the study on variation in the final pH of the hydrolysates allowed us to elucidate the effect on their antimicrobial and antioxidant activities, as well as their potential food applications according to this variable. It was established that a higher pH showed stronger anti-yeast activity and lower DPPH-scavenging capacity, while pH 7 favored antifungal activity. This supports the influence of pH on the mechanisms involved in the inhibition of microorganisms and oxidative chain reactions. According to our results, the use of PEF treatments could be a suitable alternative to conventional methods of pepsin inactivation (chemical or thermal inactivation) during the production of bioactive peptides via enzymatic hydrolysis, by reducing the chemical reagents or energy consumption. This could represent an improvement in the eco-efficiency of this process. To support these promising results, further studies are suggested for the optimization of the conditions for pepsin inactivation, such as electric-field strength, frequency, flow rate, operating time, number of pulses, etc. Likewise, PEF treatments could potentially be used to produce discolored hemoglobin hydrolysates in a more eco-efficient way, which are conventionally prepared by precipitating the heme groups via acidification. Indeed, the inactivation of pepsin by PEFs allows for stopping of the enzymatic reaction at an acidic pH, and hence, this step could be used to promote hemoglobin precipitation without additional acid consumption.

## Figures and Tables

**Table 1:** Antimicrobial activity of Hb-B or Hb-P hydrolysates (40 mg/mL) at different pH values (3, 7, and 10) produced by pepsin after 3 h of hydrolysis.

Treatment	Diameter of Growth Inhibition (mm)							
	Hb-B				Hb-P			
	*E. coli* MP 4100	*L. ivanovii* HP B28	*M. racemosus* LMA-722	*R. mucilaginosa* 27,173	*E. coli* MP 4100	*L. ivanovii* HP B28	*M. racemosus* LMA-722	*R. mucilaginosa* 27,173
PEF-pH 3	-	7.7 ± 0.6 ^b^	-	9.7 ± 1.2 ^d^	-	7.7 ± 0.6 ^c^	-	17.3 ± 0.6 ^b^
PEF-pH 7	-	8.3 ± 1.5 ^b^	9.7 ± 0.6 ^cd^	15.3 ± 0.6 ^b^	-	10.7 ± 0.6 ^b^	10.3 ± 0.6 ^bc^	20.7 ± 1.2 ^a^
PEF-pH 10	-	8.3 ± 0.6 ^b^	11.7 ± 0.6 ^ab^	15.3 ± 0.6 ^b^	-	10.7 ± 0.6 ^b^	14.3 ± 0.6 ^a^	21.7 ± 0.6 ^a^
C-pH 3	-	7.3 ± 0.6 ^b^	-	15.3 ± 0.6 ^b^	-	7.7 ± 0.6 ^c^	-	21.0 ± 1.0 ^a^
C-pH 7	-	7.7 ± 0.6 ^b^	10.7 ± 0.6 ^bc^	17 ± 0.0 ^ab^	-	10.7 ± 0.6 ^b^	12.0 ± 0.0 ^b^	21.7 ± 0.6 ^a^
C-pH 10	-	8.3 ± 0.6 ^b^	12.7 ± 0.6 ^a^	17.7 ± 0.6 ^a^	-	10.7 ± 0.6 ^b^	15.3 ± 0.6 ^a^	22.0 ± 0.0 ^a^
Hb pH3	-	-	-	12 ± 0.0 ^c^	-	-	-	11.0 ± 0.0 ^c^
Hb pH7	-	-	-	-	-	-	-	-
Hb pH10	-	-	-	-	-	-	-	-
Solution pH3	-	-	-	-	-	-	-	-
Solution pH7	-	-	-	-	-	-	-	-
Solution pH10	-	-	-	-	-	-	-	-
Ampicillin	22.3 ± 0.8	27.7 ± 0.6 ^a^	-	-	21.5 ± 1	27.5 ± 0.5 ^a^	-	-
Natamycin	-	-	9.2 ± 0.8 ^d^	9.8 ± 0.8 ^d^	-	-	8.8 ± 1.2 ^d^	10.5 ± 0.5 ^c^

Hb: native hemoglobin. Means and standard deviations were calculated from three repetitions. Means in columns followed by the same letter are not significantly different according to Tukey test at the 5% level.

**Table 2:** Determination of MIC, MBC/MIC, and MFC/MIB of Hb-B hydrolysates or Hb-P hydrolysates at different pH values (3, 7, and 10) produced by pepsin after 3 h of hydrolysis.

Source	Treatment	*E. coli* MP 4100			*L. ivanovii* HP B28			*M. racemosus* LMA-722			*R. mucilaginosa* 27,173		
		MIC (mg/mL)	MBC (mg/mL)	MBC/MIC	MIC (mg/mL)	MBC (mg/mL)	MBC/MIC	MIC (mg/mL)	MFC (mg/mL)	MFC/MIC	MIC (mg/mL)	MFC (mg/mL)	MFC/MIC
Hb-B	PEF-pH 3	-	-	-	5.00 ± 0.00 ^a^	20.00 ± 0.00 ^b^	4.00 ± 0.00	-	-	-	10.00 ± 0.00 ^b^	20.00 ± 0.00 ^b^	2.00 ± 0.00
	PEF-pH 7	-	-	-	5.00 ± 0.00 ^a^	>20.00 ± 0.00 ^a^	>8.00 ± 0.00	5.00 ± 0.00 ^b^	5.00 ± 0.00 ^b^	1.00 ± 0.00	1.25 ± 0.00 ^e^	2.50 ± 0.00 ^e^	2.00 ± 0.00
	PEF-pH 10	-	-	-	5.00 ± 0.00 ^a^	>20.00 ± 0.00 ^a^	>8.00 ± 0.00	>20.00 ± 0.00 ^a^	>20.00 ± 0.00 ^a^	1.00 ± 0.00	0.63 ± 0.00 ^f^	1.25 ± 0.00 ^f^	2.00 ± 0.00
	C-pH 3	-	-	-	5.00 ± 0.00 ^a^	20.00 ± 0.00 ^b^	4.00 ± 0.00	-	-	-	2.50 ± 0.00 ^d^	5.00 ± 0.00 ^d^	2.00 ± 0.00
	C-pH 7	-	-	-	5.00 ± 0.00 ^a^	>20.00 ± 0.00 ^a^	>8.00 ± 0.00	5.00 ± 0.00 ^b^	5.00 ± 0.00 ^b^	1.00 ± 0.00	1.25 ± 0.00 ^e^	2.50 ± 0.00 ^e^	2.00 ± 0.00
	C-pH 10	-	-	-	5.00 ± 0.00 ^a^	>20.00 ± 0.00 ^a^	>8.00 ± 0.00	>20.00 ± 0.00 ^a^	>20.00 ± 0.00 ^a^	1.00 ± 0.00	0.63 ± 0.00 ^f^	0.63 ± 0.00 ^g^	1.00 ± 0.00
	Hb-pH3	-	-	-	-	-	-	-	-	-	5.00 ± 0.00 ^c^	5.00 ± 0.00 ^d^	1.00 ± 0.00
	Hb-pH7	-	-	-	-	-	-	-	-	-	-	-	-
	Hb-pH10	-	-	-	-	-	-	-	-	-	-	-	-
Hb-P	PEF-pH 3	-	-	-	2.50 ± 0.00 ^b^	20.00 ± 0.00^b^	8.00 ± 0.00	-	-	-	5.00 ± 0.00 ^c^	10.00 ± 0.00 ^c^	2.00 ± 0.00
	PEF-pH 7	-	-	-	2.50 ± 0.00 ^b^	>20.00 ± 0.00^a^	>16.00 ± 0.00	1.25.00 ± 0.00 ^c^	1.25.00 ± 0.00 ^c^	1.00 ± 0.00	0.62 ± 0.00 ^f^	0.62 ± 0.00 ^g^	1.00 ± 0.00
	PEF-pH 10	-	-	-	2.50 ± 0.00 ^b^	>20.00 ± 0.00^a^	>16.00 ± 0.00	1.25.00 ± 0.00 ^c^	1.25.00 ± 0.00 ^c^	1.00 ± 0.00	0.63 ± 0.00 ^f^	0.63 ± 0.00 ^g^	1.00 ± 0.00
	C-pH 3	-	-	-	2.50 ± 0.00 ^b^	20.00 ± 0.00^b^	8.00 ± 0.00	-	-	-	0.63 ± 0.00 ^f^	0.63 ± 0.00 ^g^	1.00 ± 0.00
	C-pH 7	-	-	-	2.50 ± 0.00 ^b^	>20.00 ± 0.00^a^	>16.00 ± 0.00	1.25.00 ± 0.00 ^c^	1.25.00 ± 0.00 ^c^	1.00 ± 0.00	0.63 ± 0.00 ^f^	0.63 ± 0.00 ^g^	1.00 ± 0.00
	C-pH 10	-	-	-	2.50 ± 0.00 ^b^	>20.00 ± 0.00^a^	>16.00 ± 0.00	1.25.00 ± 0.00 ^c^	1.25.00 ± 0.00 ^c^	1.00 ± 0.00	0.31 ± 0.00 ^g^	0.62 ± 0.00 ^g^	2.00 ± 0.00
	Hb pH3	-	-	-	-	-	-	-	-	-	5.00 ± 0.00 ^c^	5.00 ± 0.00 ^d^	1.00 ± 0.00
	Hb pH7	-	-	-	-	-	-	-	-	-	-	-	-
	Hb pH10	-	-	-	-	-	-	-	-	-	20.00 ± 0.00 ^a^	>20.00 ± 0.00 ^a^	2.00 ± 0.00
Ampicillin	Antibiotic (C+)	0.016 ± 0.000	0.032 ± 0.000	0.002 ± 0.000	0.004 ± 0.000 ^c^	0.016 ± 0.000 ^c^	0.004 ± 0.000	-	-	-	-	-	-
Natamycin	Antifungal (C+)	-	-	-	-	-	-	0.004 ± 0.000 ^d^	0.004 ± 0.000 ^d^	0.001 ± 0.000	0.004 ± 0.000 ^h^	0.008 ± 0.000 ^h^	0.002 ± 0.000

Hb: native hemoglobin. MIC: minimum inhibitory concentration. MBC: minimum bactericidal concentration. MFC: minimum fungicidal concentration. Means and standard deviations were calculated from three repetitions. Means in columns followed by the same letter are not significantly different according to Tukey test at the 5% level.

**Table 3:** Comparison of antioxidant activity prediction of common potential antioxidant peptides identified in hydrolysates of Hb-B and Hb-P by AnOxPePred algorithm and Basic Local Alignment Search Tool (BLAST).

Hb-B Hydrolysates			Hb-P Hydrolysates			Query Cover (%)	Identity (%)
Location	Sequence	FRS Score	Location	Sequence	FRS Score		
β(31–40)	LVVYPWTQRF	0.574	β(33–42)	LVVYPWTQRF	0.574	100	100
β(32–40)	VVYPWTQRF	0.571	β(34–42)	VVYPWTQRF	0.571	100	100
β(32–37)	VVYPWT	0.569	β(34–39)	VVYPWT	0.569	100	100
β(140–145)	LAH**R**YH	0.565	β(142–147)	LAH**K**YH	0.541	100	83.3
β(138–145)	NALAH**R**YH	0.523	β(140–147)	NALAH**K**YH	0.493	100	87.5
α(37–46)	PTTKTYFPHF	0.512	α(37–46)	PTTKTYFPHF	0.512	100	100
α(34–46)	L**S**FPTTKTYFPHF	0.433	α(34–46)	L**G**FPTTKTYFPHF	0.459	100	92.3
α(110–125)	A**S**H**L**P**S**DF**T**P**A**VHASL	0.431	α(110–125)	A**A**H**H**P**D**DF**N**P**S**VHASL	0.428	100	68.8
α(138–141)	SKYR	0.429	α(138–141)	SKYR	0.429	100	100
β(135–145)	GVANALAH**R**YH	0.428	β(137–147)	GVANALAH**K**YH	0.432	100	90.9
α(47–53)	**D**LSHGS**A**	0.413	α(47–53)	**N**LSHGS**D**	0.434	85	83.3
β(96–102)	HVDPENF	0.426	β(98–104)	HVDPENF	0.426	100	100
β(27–29)	LG**R**	0.416	α(34–36)	LG**F**	0.425	nd	nd
β(130–145)	QKVVAGVANALAH**R**YH	0.427	β(132–147)	QKVVAGVANALAH**K**YH	0.416	100	93.8
α(66–80)	LTKAV**E**HLDDLPGAL	0.398	α(66–80)	LTKAV**G**HLDDLPGAL	0.412	100	93.3
β(96–101)	HVDPEN	0.411	β(98–103)	HVDPEN	0.411	100	100
α(65–80)	ALTKAV**E**HLDDLPGAL	0.411	α(65–80)	ALTKAV**G**HLDDLPGAL	0.425	100	93.8
α(109–125)	LA**S**H**L**P**S**DF**T**P**A**VHASL	0.393	α(109–125)	LA**A**H**H**P**D**DF**N**P**S**VHASL	0.405	100	70.6
α(84–98)	SDLHAHKLRVDPVNF	0.388	α(84–98)	SDLHAHKLRVDPVNF	0.388	100	100
α(137–141)	TSKYR	0.384	α(137–141)	TSKYR	0.384	100	100

FRS: free-radical scavenging. nd: not determined. FRS scores > 0.43 provide the best cut-off between positive and negative predictions according to the models. Bold script represents the different amino acids between the peptide sequences. Query cover indicates the percentage of the query length that is included in the aligned segments.

## Data Availability

Data is contained within the article or supplementary materials.

## Supplementary Materials

The following supporting information can be downloaded at: https://www.mdpi.com/article/10.3390/foods11213313/s1, Table S1: Predicted values of free-radical-scavenging (FRS) score for Hb-B hydrolysates; Table S2: Predicted values of free-radical-scavenging (FRS) score for Hb-P hydrolysates.
